# Handling of Ventricular Fibrillation in the Emergency Setting

**DOI:** 10.3389/fphar.2019.01640

**Published:** 2020-01-29

**Authors:** Zoltán Szabó, Dóra Ujvárosy, Tamás Ötvös, Veronika Sebestyén, Péter P. Nánási

**Affiliations:** ^1^ Department of Emergency Medicine, Faculty of Medicine, University of Debrecen, Debrecen, Hungary; ^2^ Doctoral School of Health Sciences, Faculty of Public Health, University of Debrecen, Debrecen, Hungary; ^3^ Department of Physiology, Faculty of Medicine, University of Debrecen, Debrecen, Hungary; ^4^ Department of Dental Physiology, Faculty of Dentistry, University of Debrecen, Debrecen, Hungary

**Keywords:** ventricular fibrillation, sudden cardiac death, cardiopulmonary resuscitation, ventricular repolarization, electrocardiography

## Abstract

Ventricular fibrillation (VF) and sudden cardiac death (SCD) are predominantly caused by channelopathies and cardiomyopathies in youngsters and coronary heart disease in the elderly. Temporary factors, e.g., electrolyte imbalance, drug interactions, and substance abuses may play an additive role in arrhythmogenesis. Ectopic automaticity, triggered activity, and reentry mechanisms are known as important electrophysiological substrates for VF determining the antiarrhythmic therapies at the same time. Emergency need for electrical cardioversion is supported by the fact that every minute without defibrillation decreases survival rates by approximately 7%–10%. Thus, early defibrillation is an essential part of antiarrhythmic emergency management. Drug therapy has its relevance rather in the prevention of sudden cardiac death, where early recognition and treatment of the underlying disease has significant importance. Cardioprotective and antiarrhythmic effects of beta blockers in patients predisposed to sudden cardiac death were highlighted in numerous studies, hence nowadays these drugs are considered to be the cornerstones of the prevention and treatment of life-threatening ventricular arrhythmias. Nevertheless, other medical therapies have not been proven to be useful in the prevention of VF. Although amiodarone has shown positive results occasionally, this was not demonstrated to be consistent. Furthermore, the potential proarrhythmic effects of drugs may also limit their applicability. Based on these unfavorable observations we highlight the importance of arrhythmia prevention, where echocardiography, electrocardiography and laboratory testing play a significant role even in the emergency setting. In the following we provide a summary on the latest developments on cardiopulmonary resuscitation, and the evaluation and preventive treatment possibilities of patients with increased susceptibility to VF and SCD.

## Introduction

Ventricular fibrillation (VF) is an emergency condition that, without immediate treatment, leads to death. In the event of this malignant ventricular arrhythmia chaotic, disorganized electrical activity appears in the ventricular myocardium. In such cases the heart is unable to transport blood effectively, which leads to circulatory collapse, clinical death, and if left unattended, biological death. Sudden cardiac death (SCD) is a leading death cause all over the world, affecting some 5 million people a year (Berdowski et al., 2010; Glinge et al., 2016) with a low survival rate of approximately 10% (Kim et al., 2016). In the United States 300–400 thousand, while in Europe 700 thousand cases of sudden cardiac death are registered a year. Its incidence is 100/100000 in the case of 50-year-old men, it is as high as 800/100000 among men 75 years of age. SCD is more frequent among men than women (6.68/100000 vs. 1.4/100000) (Eckart et al., 2011; Priori et al., 2015). VF is the arrhythmia to manifest first and with the highest frequency in relation to registered circulatory collapses (Rea et al., 2004), which accounts for about 30% of the cases (Bourque et al., 2007).

## Pathological Conditions Underlying Ventricular Fibrillation

The most common factors that may contribute to the development of VF are acquired risk factors, e.g., ischemic heart disease, cardiomyopathies, electrolyte imbalances, certain drug therapies affecting myocardial repolarization, and less frequently inherited disorders, e.g., ion channel abnormalities (channelopathies), congenital valvular diseases, and coronary artery anomalies. (**Table 1**) (Garson and McNamara, 1985; Jouven et al., 1999; Thompson et al., 2000; Frothingham, 2001; Haddad and Anderson, 2002; Taylor, 2003; Yap and Camm, 2003; Vieweg and Wood, 2004; Owens and Ambrose, 2005; Haissaguerre et al., 2008; Priori et al., 2015; Aiba, 2019; Coutinho Cruz et al., 2019).

**Table 1:** Etiology of ventricular fibrillation.

PATHOLOGICAL FACTORS THAT PLAY A ROLE IN THE DEVELOPMENT OF VENTRICULAR FIBRILLATION		
INHERITED	ACQUIRED	TEMPORARY CAUSES
long QT syndrome	ischemic heart disease	sepsis
short QT syndrome	chronic kidney disease	electrolyte disorders (especially K^+^ and Mg^++^)
Brugada-syndrome	hypertension	electrocution
catecholaminergic polymorphic ventricular tachycardia	cardiomyopathies	drug abuse (cocaine, methamphetamine)
arrhythmogenic right ventricular dysplasia	acquired aortic disorders	provocative body posture in the case of structural heart disease
single nucleotide polymorphisms (e.g. on 21q21 and 2q24.2 loci)	prior aborted cardiac death	drugs affecting QT interval: antimicrobial agents antipsychotics antidepressants antiarrhythmic drugs: amiodarone, sotalol, procainamide, quinidine, dofetilide, ibutilide
J-point elevation syndromes	post-valvular surgery, post-TGA surgery or other heart surgeries	
inherited structural heart diseases (tetralogy of Fallot, VSD, mitral prolapse, aortic disorders)	post-PCI or post-thrombolysis (reperfusion damage)	

VSD, ventricular septal defect, TGA, transposition of the great arteries, PCI, primary coronary intervention.

Channelopathies listed under inherited primary arrhythmia syndromes, like long and short QT syndromes and Brugada-syndrome (BrS), are important pathogenetic factors (Aiba, 2019). In 70% of long QT syndromes (LQTS) the mutation of the *KCNQ1* or the *KCNH2* (*hERG*) genes have been clarified. These genes encode the potassium channels responsible for the slow (I_Ks_) and rapid component (I_Kr_) of the delayed rectifier repolarising potassium current, respectively. Beyond *KCNQ1* (LQT1) and *KCNH2* (LQT2), *SCN5A* (LQT3) is the third most frequently affected gene that plays a role in the genesis of LQTS. Mutation of the *SCNA5* contributes to an increase in the depolarising sodium inward current leading to the consequent prolongation of the QT interval (Priori et al., 2013). Furthermore, the role of mutations of the L-type calcium channel and other structural proteins was described in the pathogenesis of LQTS, however the triggering mutation has not been identified yet in many other cases (Schwartz et al., 2012). In the case of catecholaminergic polymorphic ventricular tachycardia (CPVT) the autosomal dominant inherited mutation of the type-2 cardiac ryanodine receptor has been identified as a possible cause. Moreover, the mutation of the Kir2.1 inward rectifier potassium channel encoded by *KCNJ2* gene can also lead to CPVT (Priori et al., 2013). The Brugada syndrome is known as a familial disease which is inherited in an autosomal dominant pattern with incomplete penetration, however in 60% of the cases occurs sporadically (Campuzano et al., 2010). Nearly 400 gene mutations have been described as a possible underlying factor in the genesis of this disease, nevertheless, in more than 80% of the cases the mutation and/or copy number variation of *SCN5A* gene encoding the Kv1.5 voltage gated sodium channel have been found (Kapplinger et al., 2010; Eastaugh et al., 2011). However, recently a thorough evaluation of the routine genetic testing panels was made. As a result, mutation of *SCNA5* gene was proven to be the only real, clinically valid disease-causing gene in case of BrS. Other gene-disease associations appeared to be debatable (Hosseini et al., 2018). The highest occurence of ECG alterations and cardiac events were reported in case of *SCNA5* mutation among BrS patients (Yamagata et al., 2017). Several gene polymorphisms, epigenetic factors and posttranslational modifications were also revealed as a pathogenic condition of BrS in the past few years (Kim et al., 2013; Webster et al., 2013) Arrhythmogenic right ventricular dysplasia (ARVD) may also have an effect in arrhythmogenesis and the development of SCD (Marcus et al, 2010). The single nucleotide polymorphisms (SNPs) located in the 21q21 and 2q24.2 loci also contribute to an increased risk for SCD (Bezzina et al., 2010; Arking et al., 2011).

## The Electrophysiological Mechanisms Underlying the Development of Ventricular Fibrillation

The electrical (repolarization) heterogeneity and secondary anisotropy of the ventricular myocardium play a crucial role in the genesis of VF. The monophasic action potentials (MAPs) characterising the electrical feature of a myocardial cell in the endocardial, mid-myocardial, and epicardial layers differ from each other, where types and expression levels of potassium channels responsible for transient outward currents (I_to_) are also different. These dissimilarities can form a transmural voltage gradient during the activation of the ventricular myocardium. As a result, increased ventricular electrical anisotropy and an enhanced ventricular arrhythmia vulnerability may appear (Antzelevitch et al., 2000). Anisotropy can also be influenced by the differences in the distribution of intercellular electrical signal transducer gap junctions (Rohr, 2004). The macroscopic discontinuity of the myocardium (e.g., presence of septa, trabecula, papillary muscles), and the accumulation of connective tissue due to hypertrophy or infarction may also generate ventricular anisotropy (Antzelevitch et al., 2000). Scars and fibrotic tissues deposited parallel (not perpendicular) to the myocardial fibers can cease intercellular connections, consequently electrical conduction may turn irregular (Spach and Josephson, 1994). In the case of myocardial hypertrophy and heart failure (HF) the prolongation of action potential duration (APD) may consequently form early afterdepolarizations (EADs) (Nabauer and Kaab, 1998) Heart failure can cause a significant decrease in the repolarising I_to_ and delayed rectifier potassium currents (I_Kr_ and I_Ks_ currents of K channels encoded by *KCNH2* and *KCNQ1* genes) that may enhance the effect of proarrhythmic factors (e.g., hypokalemia, hypomagnesemia, class III antiarrhythmic drugs) (Nuss et al., 1996; Anumonwo and Lopatin, 2010). In the case of LQTS the mutation of potassium channels decreases their function by at least 50% leading to a prolonged repolarization, APD and QT interval. Class III antiarrhythmic drugs have a similar APD prolonging effect, that may contribute to an increased ventricular proarrhythmic susceptibility (Nuss et al, 1996; Peters et al., 2000). In the cases of heart failure and left ventricular hypertrophy, the downregulation of potassium channels, the decrease of transient outward currents (I_to_) and the decrease of rapid and slow components of the delayed rectifier currents (I_Kr_, I_Ks_) can contribute to the prolongation of APD. Regarding HF, the overexpression of *HCN2* and *HCN4* genes can appear resulting in an increased pacemaker current (I_f_) not only in the SA node, but in the ventricular tissue, too. The electrical remodeling of the heart can lead to a reduced ventricular repolarization reserve, increasing the risk of ventricular arrhythmias based on triggered activity, ectopic automaticity, and reentry mechanisms (Cerbai et al., 2001; Stillitano et al., 2008). ATP-sensitive potassium channels (K_ATP_ channels) localized in the sarcolemmal membrane may be activated by metabolic stress or ischemia causing the shortening of APD, preventing the heart from calcium overload and contraction abnormalities. Moreover, myocardial hypertrophy and ischemia can increase the expression levels of Kir 6.1 and Kir 6.2 K_ATP_ channels, leading to an increased risk for ventricular dysrhythmias based on heterogeneous shortening of the AP (Akrouh et al., 2009; Tinker et al., 2018). In 1998 repolarization reserve has been defined by Roden et al. as a defensive mechanism of the myocardium, which protects the heart from the emergence of arrhythmias in particular circumstances where APD has already been prolonged. For instance, enhanced inward Na^+^ currents contributes to an increase in delayed rectifier outward potassium current (especially I_Kr_) as a compensatory mechanism in order to prevent the prolongation of repolarization. Furthermore, congenital alterations in ion currents of the myocardium, heart failure, cardiac hypertrophy, bradycardia, increased sympathetic activation, female gender, recent pharmacological conversion from atrial fibrillation to sinus rhythm, use of diuretics, hypokalemia, hypomagnesemia, administration of class IA, IC or III antiarrhythmic drugs or agents with I_Kr_ blocking effect can decrease repolarization reserve. Co-occurence of certain conditions which affect more than one type of ion currents involved in regular repolarization may critically reduce repolarization reserve and can result in proarrhythmia (Roden, 1998; Varró and Baczkó, 2011).

**Figure 1:**
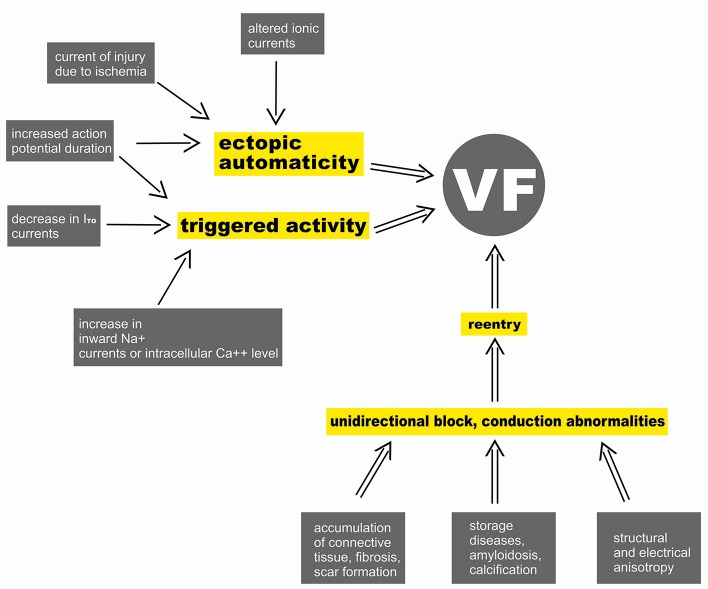
Pathomechanism of ventricular fibrillation (VF).

The underlying electrophysiological mechanisms of ventricular fibrillation consequently include *ectopic automaticity*, *reentry* and *triggered activity* (due to early and late afterdepolarizations) (**Figure 1**) (Bayes De Luna et al., 1989; Peters et al., 2000).

*Ectopic automaticity* is the result of spontaneous diastolic depolarization, where current of injury (local alterations in K^+^ gradient) due to acute ischemic damage is an important provoking mechanism (Thygesen and Uretsky, 2004). In the case of *ectopic automaticity* it is a ventricular premature beat caused by inherited or acquired ectopic impulse-generating foci that most frequently play a role in triggering VF (Van Camp, 1992; Singh and Noheria, 2018). Ventricular premature beats may arise from any part of the electrical conducting system of the heart, especially from the Purkinje fibers, and may also originate from the right or left ventricular outflow tract, or the papillary muscles. Nevertheless, monomorphic ventricular tachycardia or atrial fibrillation as part of a preexcitation syndrome may also contribute to VF (Viskin et al., 2013).

A frequent underlying cause of the unidirectional block serving as the basis of *reentry mechanism* may be the prolongation of the monophasic action potential of the myocyte and the consequential heterogeneity of ventricular repolarization. This process may be facilitated by myocardial ischemia and may trigger an increase in the monophasic AP duration restitution slope as well as changes in the amplitude of AP (electrical alternans) (Krummen et al., 2016).

*Triggered activity* may also appear as a consequence of early (EAD) or delayed afterdepolarization (DAD). EAD is caused by the early reactivation of L-type Ca-channels, a consequence of a decrease in repolarising potassium currents or an increase in the activity of positive currents toward the intracellular space (Roden, 2016). Furthermore, oxidative stress, hypokalemia may also play an additive role in the pathomechanism of EAD (Antzelevitch et al., 1991; Karagueuzian et al., 2013; Antzelevitch et al., 2014; Markandeya and Kamp, 2015). In contrast, DAD develops after the repolarization of the myocyte membrane due to intracellular calcium excess or an increased sensitivity of intracellular ryanodine receptors (Priori and Chen, 2011). Delayed after depolarizations often serve as a background for ventricular arrhythmias caused by heart failure or digoxin toxicity and can also play a role in the genesis of CPVT (Priori and Chen, 2011).

## Risk Assessment of Ventricular Fibrillation

Several diagnostic tools are available for the risk stratification of ventricular arrhythmias and sudden cardiac death. Certain parameters of the 12-lead surface ECG (e.g., QT interval and dispersion, T wave peak-to-end interval, arrhythmogeneity index, epsilon wave, delta wave, Brugada sign, J point elevation, etc.) may be of help in predicting life-threatening ventricular dysrhythmias and clarifying the underlying arrhythmia mechanism. Holter ECG may be useful in arrhythmia risk stratification (by determining T wave alternans, heart rate variability, QT variability, etc.). Echocardiography is widely used to determine structural and functional abnormalities of the heart. Accumulation of connective tissue in the left ventricle (scar burden) and fatty tissue in the right ventricle (ARVD) can be examined by MRI. Coronary CT angiography and coronarography may be of help to recognize coronary artery disease as a possible underlying arrhythmogenic factor. Electrophysiological testing is suitable for the examination of VT/VF inducibility and for the definition of the arrhythmia foci. Serum level of certain biomarkers may also be taken into consideration as part of ventricular arrhythmia risk assessment (Deyell et al., 2015).

### Laboratory Opportunities for Predicting Ventricular Arrhythmias

Considering laboratory biomarkers, in addition to the now more widely used NT-proBNP and the high-sensitive troponin T, osteopontin, galectin-3, and soluble ST2 can also be applied to determine the arrhythmia vulnerability of the ventricular myocardium (Pascual-Figal et al., 2009; Patton et al., 2011; Francia et al., 2014; Xu et al., 2019). NT-proBNP is a neurohormone produced by the brain, the left atrium, and the left ventricle, whose serum level significantly rises in the case of left ventricular dysfunction. Examining the population of the Cardiovascular Health Study Patton et al. found 289 cases of sudden cardiac death where the increased NT-proBNP value involved 4.2 times higher risk of the development of sudden cardiac death (Patton et al., 2011). Xu et al. examined 104 patients treated with aborted cardiac death where during the follow-up period a higher hsTnT level was measured in the case of patients who suffered repeated episodes of ventricular fibrillation (Xu et al., 2019). Osteopontin and galectin-3 are primarily ventricular myocardium-specific fibrosis markers whose increased serum-level implies enhanced myocardial fibrosis and thereby, due to the increased electrical heterogeneity, enhanced risk of ventricular arrhythmias (Francia et al., 2014). Soluble ST2 is a member of the interleukin-1 receptor family, produced by cardiomyocytes. Pascual-Figal et al. found that increased sST2 values involved 1.39 times higher risk of sudden cardiac death compared to persons with a regular serum level (Pascual-Figal et al., 2009). In a cohort study based on the clinical data of 72 patients, Scheirlynck et al. proved that mitral annulus disjunction (MAD) was linked to a higher sST2 level in the presence of ventricular arrhythmias. The focal fibrosis of the mitral apparatus has been shown to be associated with the electrical instability and the hypermobility of the mitral valve, which together can lead to an enhanced ventricular arrhythmia susceptibility. In contrary to the study of Patton et al., they found normal NT-proBNP and CRP levels in their population. In the same study TGFß1 was assumed to be responsible for the ventricular arrhythmogeneity (Scheirlynck et al., 2019). In the Physicians’ Health Study a positive correlation was reported between high serum CRP levels and increased arrhythmia risk vulnerability, however in the Nurse’s Health Study population similar connection could not be proven (Balla et al., 2019). In the Paris study, the increased serum concentration of non-esterified free fatty acids were described as an independent risk factor for SCD in middle-aged men (Balla et al., 2019). Furthermore, increased serum levels of matrix metalloproteinazes (MMPs) and their inhibitor molecules (TIMPs) - which may take part in the genesis of cardiac fibrosis - have also been shown to contribute to an increased risk of ventricular dysrhythmias (Klappacher et al., 1995). In another investigation by Benito et al. the elevated serum level of testosterone, and the decreased serum level of oestrogen were in correlation with an increased probability of malignant ventricular arrhythmias (Benito et al., 2008).

### Estimating Arrhythmia Susceptibility With Imaging Methods

Left ventricular ejection fraction (EF) measured during echocardiography is suitable for predicting malignant ventricular arrhythmia and, according to current knowledge, is the only echocardiographic parameter with predictive value for an increased risk of sudden cardiac death in the case of ischemic heart disease and left ventricular dysfunction (Fogel, 2002; Moss et al., 2002; Alberte and Zipes, 2003). Echocardiographic examinations may also be of help in the detection of structural heart diseases (concentric and eccentric left ventricular hypertrophy, valvulopathies, chamber diameters, etc.) underlying ventricular arrhythmias (Cheitlin et al., 2003; Priori et al., 2015).

Another opportunity is to perform a cardiac MR, which thanks to its high resolution gives a more precise picture of wall motion disorders, is suitable for performing more refined volumetric measurements and provides a better approximation of the left ventricular mass than echocardiography, is at the same time much more expensive, less accessible, and may requires the administration of a contrast agent. It is of outstanding value in the diagnostics of patients with arrhythmogenic right ventricular dysplasia, in assessing the tissue structure of the ventricular wall (Priori et al., 2015).

### Electrocardiographic Estimation of the Risk for Ventricular Arrhythmias

Determining the respective parameters of a 12-lead surface ECG is an inexpensive, easy to perform and reproducable method, which can be widely used in the everyday clinical practice for predicting ventricular arrhythmias.

*Short QT interval* (QT interval corrected to heart rate <300 ms) and *long QT interval* (QT interval corrected to heart rate, for men ≥ 451 ms, for women ≥471 ms) involve a confirmed increased risk of sudden cardiac death (Malik and Batchvarov, 2000; Boriani et al., 2006). Zhang et al. showed that the relative risk of SCD comparing patients with the longest and shortest QT interval was 1.44 (Zhang et al., 2011). Vink et al. proved that manual measurement of QT interval corrected according to Bazett formula has a specificity of 86% and a sensitivity of 85% simultaneously (Vink et al., 2018). QT dispersion is also a widely used ventricular arrhythmia risk marker; it is the difference between the longest and shortest QT intervals in the 12-lead electrocardiogram and can be used to estimate the spatial dispersion of ventricular repolarization. Increased QT dispersion, showing a repolarization heterogeneity, has been reported in several pathological conditions; its predictive value for an arrhytmia risk and SCD has been confirmed in the cases of amyloidosis, hyperlipidemia, systemic sclerosis, thyroid dysfunction, diabetes mellitus, and chronic kidney disease (Sgreccia et al., 1998; Lőrincz et al., 1999; Cardoso et al., 2001; Szabó et al., 2005; Bakiner et al., 2008; Gilotra et al., 2013; Tse and Yan, 2017). Interestingly, when compared to hemodiafiltration QT dispersion has been found to be significantly higher during hemodialysis (Barta et al., 2014). In the case of patients with coronary artery disease increased QT dispersion had 92% sensitivity and 81% specificity in the prediction of SCD (Darbar et al., 1996).

Short-term beat-to-beat variability of the QT interval (QTv) can be determined manually or by means of a computer software. Generally, 30 consecutive QT intervals in leads II and V5 are measured (Berger et al., 1997). Increased QTv has been described in patients with dilated cardiomyopathy (DCM), heart failure, drug-induced or congenital long QT syndromes, panic disorder, and even in the cases of young athletes with moderate left ventricular hypertrophy (Hinterseer et al., 2008; Hinterseer et al., 2009; Hinterseer et al., 2010; Lengyel et al., 2011; Varkevisser et al., 2012). Furthermore, QTv was shown to be useful in the detection of latent repolarization disorders. QT variability index (QTVI) can be calculated from the logarithmic ratio of the mean QTc interval and heart rate and the variability of QT interval and heart rate. Its increase indicates repolarization heterogeneity. QTVI may increase in many conditions, e.g., DCM, acute ischemia, HF, LQTS, renal failure, ventricular arrhythmias, and SCD (Dobson et al., 2013). However, its predictive and diagnostic value has not been clearly elucidated yet. Investigators agree that QTVI combined with QTc, QTv, and heart rate variability may improve the risk stratification of patients with enhanced arrhythmia susceptibility (Oosterhoff et al., 2011).

T wave peak-to-end interval (Tpe) and the arrhythmogeneity index (AIX, derived as the ratio of Tpe and corrected QT interval) are also suitable for estimating the danger of ventricular arrhythmias (Gupta et al., 2008; Kors et al., 2008). The normal value of Tpe in the case of men is <94 ms and for women <92 ms (Haarmark et al., 2010). The prolongation of Tpe has been observed in the case of several clinical conditions, e.g., acute myocardial infarction, sleep apnea syndrome, hypertrophic cardiomyopathy, and LQTS (Shimizu et al., 2002; Yamaguchi et al., 2003; Haarmark et al., 2009; Kilicaslan et al., 2012). In a population with liver cirrhosis Tpe value has been confirmed to have a sensitivity of 90% and a specificity of 60% in the prediction of SCD (Salgado et al., 2016). Another study with patients who underwent primary coronary intervention (PCI) showed, that the prolongation of Tpe interval with regard to SCD had a sensitivity of 90%, and a specificity of 55% with a positive predictive value of 0.18 and a negative predictive value of 0.98 (Haarmark et al., 2009). The physiological value of arrhythmogeneity index is approximately 0.19–0.2 (Zehir et al., 2015) and has proven to be even more precise than the corrected QT value in the prediction of torsades de pointes ventricular tachycardia and sudden cardiac death (Yamaguchi et al., 2003; Topilski et al., 2007; Wang et al., 2019). In another study the increase in Tpe and AIX were found to be significantly higher during hemodialysis compared to hemodiafiltration which indicates a pronounced risk for ventricular arrhythmia formation during the conventional renal replacement therapy (Páll et al., 2017). In a further investigation by Wang et al. in patients with vasospastic angina pectoris, AIX had a sensitivity of 84% and a specificity of 89.5% in the prediction of malignant ventricular arrhythmias (Wang et al., 2019).

*T wave alternans* is yet another electrocardiographic parameter that represents beat-to-beat changes in T wave morphology. Its examination may be suitable for expressing the spatial heterogeneity of ventricular repolarization (Narayan, 2006). T wave alterations measured on electrocardiogram enlarged to a microvolt scale referred to as *microvolt T wave alternans* have proven to be useful not only for estimating the risk of ventricular arrhythmia but for assessing the necessity of implantable cardioverter defibrillator (ICD) as well (Bloomfield et al., 2004; Burattini et al., 2009).

The prevalence of the *Brugada ECG*-pattern and the epsilon wave characteristic of arrhythmogenic right ventricular dysplasia both indicate an increased risk for arrhythmias (Issa et al., 2012). Priori et al. found that out of 176 Brugada-syndrome patients there were clear diagnostic signs in the resting ECG of only 90 patients (Priori et al., 2002). Therefore, in the case of clinical suspicion it is recommended to perform a provocation test using class IA or IC sodium channel blockers intravenously. Flecainide was one of the drugs that were used for this purpose, however it turned out to be ineffective in revealing the Brugada sign on the surface ECG in 30% of the examined Brugada patients (Therasse et al., 2017). Thus, ajmalin is the preferred drug with higher efficacy during provocation tests (Berne and Brugada, 2012). Procainamide can also be suitable for the triggering of not only type-1, but type-2 and -3 Brugada signs on the surface electrocardiogram. For the same purpose, disopyramide, propafenone and pilsicainide may be applied, too (Obeyesekere et al., 2011; Berne and Brugada, 2012).

Recently, growing clinical significance has been attributed to what is referred to as the J -point elevation syndrome which are the early repolarization syndromes together with Brugada syndrome (Nademanee et al., 2019). As a characteristic feature of this J-point elevation of above 0.1 mV can be measured in at least 2 related ECG leads, which manifests together with concave ST-segment elevation and peaked prominent T waves. Based on the leads with J-point alterations, 3 types of early repolarization patterns can be differentiated (**Table 2**) (Antzelevitch and Yan, 2010).

**Table 2:** Types of early repolarization syndromes (ERS) according to ECG-alterations and anatomical localization (based on Antzelevitch and Yan, 2010).

EARLY REPOLARIZATION SYNDROME (ERS)			
	TYPE 1	TYPE 2	TYPE 3
Location of J wave abnormalities (ECG leads)	I, V4-6	II, III, aVF	in all leads
Anatomical localization of repolarization disorder	the anterolateral part of the left ventricle	inferior part of left ventricle	left and right ventricle

ECG, electrocardiography.

## The Treatment of Ventricular Fibrillation

**Figure 2:**
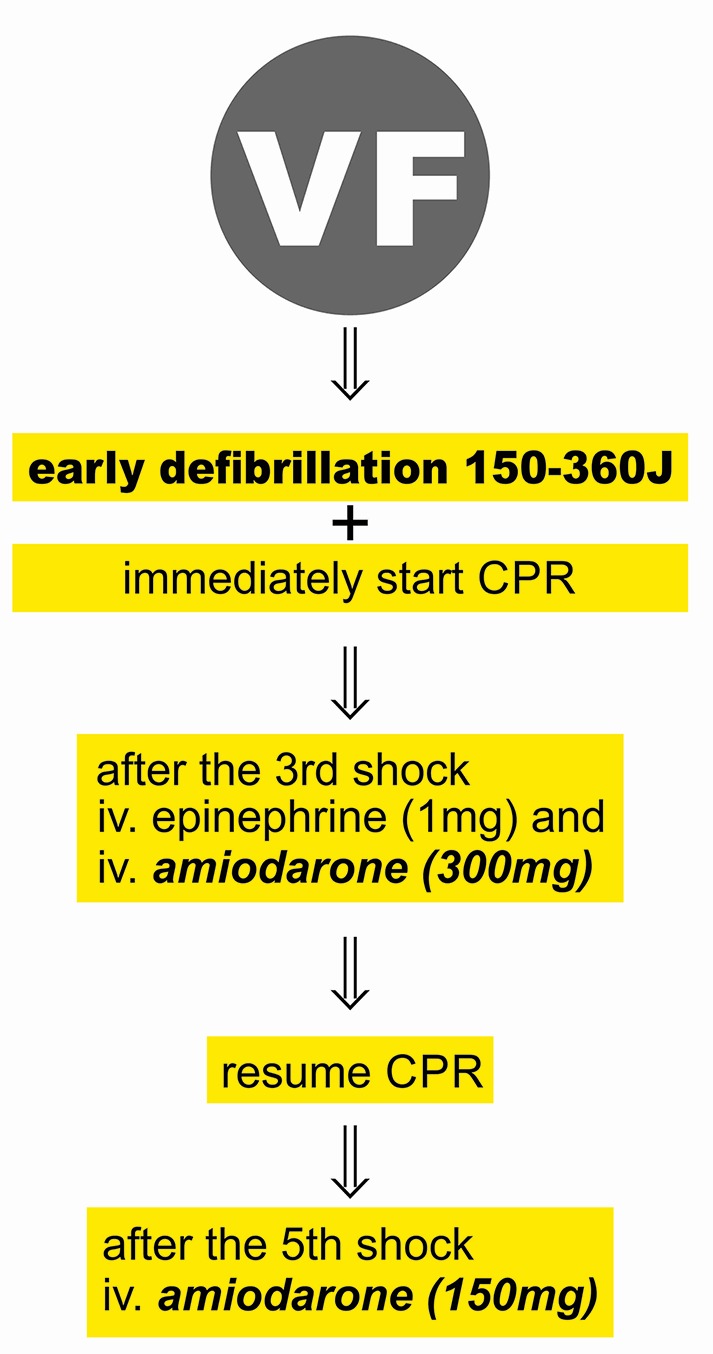
Acute treatment of ventricular fibrillation. VF, ventricular fibrillation; CPR, cardiopulmonary resuscitation.

The most effective therapies for ventricular fibrillation and pulseless ventricular tachycardia (pVT) are immediate high-quality chest compression as well as early defibrillation, which latter plays a key role in terminating these arrhythmias (**Figure 2**) (Neumar et al., 2015).

Survival rate by early defibrillation in the case of resuscitation performed by lay people is 37.4% (Benjamin et al., 2017). The necessity of earliest possible electrical treatment is underpinned by the fact that the rate of successful resuscitation falls by 7%–10% per min from the circulatory collapse (Larsen et al., 1993). The availability of automated external defibrillators (AED) in public spaces and broad-scale training for lay persons about the use of the device may increase the current survival rate of 10% (Berg et al., 2019). In Scandinavia, there have been experiments with simulation practices using drones for the speedy delivery of AEDs with positive results and applying this method in real situations in future may improve the success rate of life saving as well (Sanfridsson et al., 2019). During a practice simulating out-of-hospital circulatory collapse the results of resuscitations performed by elderly bystanders (average age: 75.5 years) were compared considering several aspects. The bystanders did not have any prior training and the primary goal was to find out how modern telecommunication tools (smartphones, drones, AED) would affect their performance. Results showed that from the time of the circulatory collapse AED was placed on the simulated patients within 10 min and resuscitation started within 2.25 min (Sanfridsson et al., 2019).

An improvement in the chance of long-term survival and the least neurological damage were found in the case when the initial rhythm observed on site was ventricular fibrillation (Daya et al., 2015). Another important element of improving the neurological outcome is post-resuscitation care started in time, which includes coronary intervention and targeted temperature management (TTM) (Link et al., 2015). Based on the examination of 136 patients, the Hypothermia after Cardiac Arrest Study Group showed that the application of TTM clearly improved neurological outcome (based on CPC) and reduced mortality (Hypothermia after Cardiac Arrest Study Group, 2002). Stanger et al. examined 570 patients resuscitated after out-of-hospital sudden cardiac death who were administered TTM therapy in hospital. The patients were put into two groups depending on how many minutes after arrival in hospital TTM was started. The greatest difference between the early (20–81 min) and the late (167–319 min) groups was in survival. The early group had 1.59 times higher chance for survival compared to the late group, while they had only 1.49 times higher chance for a good neurological outcome, which did not prove clearly significant (Stanger et al., 2019). The 2015 resuscitation guideline recommends earliest possible PCI in the case of sudden cardiac death with acute coronary syndrome as the underlying cause (Nikolaou et al., 2015). Kahn et al. had found already that early PCI performed on patients who had suffered SCD due to STEMI could help survival with a good neurological outcome (Kahn et al., 1995). Examining 35 and 190 patients respectively, Nanjayya et al. and Bro-Jeppesen et al. compared patients who underwent and others who did not undergo immediate angiography and PCI in hospital, after out-of-hospital sudden death. Both studies found that early PCI had no significant positive effect on mortality (Bro-Jeppesen et al., 2012; Nanjayya and Nayyar, 2012). In contrast, Strote et al. found by retrospectively analyzing the data of 270 patients who had suffered sudden cardiac death that acute PCI (if performed within 6 h after the emergence of symptoms) ensured significantly better survival than PCI performed beyond 6 h (Strote et al., 2012).

### Chest Compression Devices

Research findings in recent years have confirmed that a most decisive element of successful resuscitation is high-quality chest compression interrupted for the briefest possible time (Levy et al., 2015). Due to the decreasing compression depth and the increasingly frequent interruptions, the manual sustainment of good-quality chest compression recommended as a target in the guidelines faces difficulties in the short run already. In view of the latter mechanical chest compression devices have become increasingly widespread and available as alternative solutions both in prehospital and in-hospital emergency care. With the help of these devices, high-quality compressions can be maintained even for a long time (Ujvárosy et al., 2018). Although the findings of the research performed have not yet confirmed significant differences in the outcome of resuscitations with the use of these devices, there are certain situations, e.g., continuous CPR during transport, when their application is clearly recommended by international guidelines (Soar et al., 2015).

According to the international resuscitation recommendation currently in force, the European Resuscitation Council ALS protocol, the application of mechanical devices is recommended if the sustainment of high-quality chest compressions is required for a longer time, e.g. during transportation, for a hypothermic patient or in the case of PCI during ongiong CPR (Soar et al., 2015).

There are currently two major types of mechanical devices available in trade, LUCAS (Lund University Cardiopulmonary Assist System) and AutoPulse (Load distributing band CPR). LUCAS is a system operating based on a piston principle and also plays a role in the active decompression of the chest; a silicone ring is to be pressed against the patient’s chest, which thereafter exercises pressure in a depth of 5–6 cm with a frequency of 100/min, and helps the relaxation of the chest wall as well (Koster et al., 2017). In the case of AutoPulse, in contrast, a wide bandage is placed across the patient’s chest, which thereafter implements pressure on the chest with a frequency of 80/min but does not take part in the relaxation of the chest (Koster et al., 2017). Research findings comparing resuscitations performed with the respective devices have not shown any differences as regards the success of resuscitation (Gates et al., 2015).

One of the greatest doubts arising in relation to the devices was potential injuries they could cause, but as has been confirmed by several autopsy findings, mechanical devices have not caused either other types of, or more serious or frequent injuries than manual chest compression. The most frequent injury they caused was similarly rib fracture (Koster et al., 2017).

In another study in relation to resuscitation performed using a LUCAS-2 mechanical chest compression device has similarly shown favourable results. The data of altogether 287 patients were investigated who suffered out-of-hospital non-traumatic circulatory collapse, out of which resuscitation using a LUCAS-2 device was performed in 55 cases, while manual chest compression was carried out in the others. Return of spontaneous circulation (ROSC) happened in 37% of the cases; in a slightly higher rate in the mechanical group (p = 0.072). Also, in cases of prolonged resuscitation, success rate was higher in the mechanical group (p<0.05). The number of traumatic injuries was not higher in the LUCAS-2 compared to the manual group (Ujvárosy et al., 2018).

### Clinical Factors Affecting the Outcome of Resuscitation

The prognosis and long-term outcome of resuscitated patients who survived aborted cardiac death are significantly influenced by comorbidities (ischemic heart disease, hypertension), the extent of potential left ventricular hypertrophy and the mechanism of ventricular arrhythmogenesis. Koldobskiy et al. found that kidney failure, immunosuppression and obesity negatively influenced the outcome of resuscitation (Koldobskiy et al., 2014). Herlitz et al. examined the data of 33,453 patients and concluded that initial rhythm, lay resuscitation and the age of the patient very strongly influenced the outcome of CPR (Herlitz et al., 2005) Our own research findings have proven that the presence of left ventricular hypertrophy involved 5.1 times higher risk of the failure of resuscitation (p = 0.0009 r = 0.1995). We have also found that age and hypertension negatively influence success rate; in the case of hypertension there is a 1.82-time risk of a failed outcome (p = 0.018 r = 0.143) (Ujvárosy et al., 2018). Often the first and only ‘symptom’ of myocardial infarction is sudden cardiac death, and SCD was responsible for the death of almost half of the coronary patients and for nearly 325,000 deaths per year, in the USA (Myerburg et al., 1993; Singh and Noheria, 2018). Cardiological rehabilitation, favourable influence for the lipid profile, favourable medication and the management of comorbidities have nowadays significantly improved long-term prognosis in the case of SCD with ACS in the background (Bunch et al., 2005). Refractory VF, which is a relatively rare morbidity factor, involves expressly poor outcome (El-Sherif et al., 2017; Nas et al., 2019). Among the factors influencing long-term prognosis the most important one is neurological outcome, for characterising which the CPC (Cerebral Performance Category) scale is the most widely used method (**Table 3**) (Ajam et al., 2011).

**Table 3:** Cerebral performance category.

Cerebral Performance Category (CPC)	
**CPC level 1**	Normal ability or minimal disability (good cerebral performance, conscious, alert, able to work, and lead a normal life)
**CPC level 2**	Moderate cerebral disability (conscious, sufficient cerebral function for part-time work in a sheltered environment or independent activities of daily life. May have hemiplegia, seizures, ataxia, dysarthria, dysphasia, or permanent memory or mental changes).
**CPC level 3**	Severe disability (conscious, dependent on others for daily support because of impaired brain function).
**CPC level 4**	Severe disability (coma, vegetative state, no cognition, verbal or psychological interactions with the environment).
**CPC level 5**	Death (or brain death).

In addition to the application of a mechanical device, a new opportunity for prehospital emergency diagnostics available is to perform echocardiography. This may help identify some of the reversible reasons (4H-4T) causing circulatory collapse and improve the efficiency of chest compression. For the confirmation of the further roles and efficiency of echocardiography during resuscitation as well as its effect on the outcome, further studies are requried (Teran et al., 2019).

### Drug Therapy During Resuscitation

In international recommendations, therapy for VF and pVT includes the administration of epinephrine and amiodarone (Neumar et al., 2015). The mechanism of action of epinephrine in the case of cardiac arrest is the consequence to α-adrenergic effect, which, by directing systemic blood flow toward the heart increases myocardial blood supply, thus ensuring the minimum coronary perfusion pressure (CPP) required for successful defibrillation (Gough and Nolan, 2018). Proarrhythmic effect of epinephrine was described in both *ex vivo* and *in vitro* investigations. Epinephrine shortens sinus cycle length, decreases the effective refractory period of the ventricular myocardium and may also lead to increased atrial and ventricular automaticity and conduction abnormalities. Epinephrine was reported to be able to generate sinus tachycardia, supraventricular, and ventricular arrhythmias in a dose-dependent manner (Tisdale et al., 1995). Furthermore, the epicardial layer of the myocardium is more sensitive to sympathetic activation, than mid-myocardial, and the endocardial cells. Consequently, an additional heterogeneity in the APD and refractory period may appear resulting in an increased risk for ventricular arrhythmias (Antzelevitch et al., 1991; Antzelevitch and Yan, 2010). The platelet activator effect of α-adrenergic stimulation was also shown, hereby rising the possibility for microcirculatory myocardial damage (Hwang et al., 2019). Beyond its undoubtedly favorable effect with relation to cardiac arrest, its detrimental β-adrenergic activity can also be assumed. By the deterioration of systemic oxygen demand and the increase in myocardial oxygen consumption it may further aggravate the lack of balance between oxygen supply and demand (Gough and Nolan, 2018), that has a special significance in the case of ventricular fibrillation.

Inhibition of the sympathetic activity contributes to the antiarrhythmic effect of β-adrenoceptor antagonists which manifests in the prolongation of AV-nodal refractory period. By reducing Ca^++^, Na^+^ currents, and cAMP-dependent pacemaker currents (I_f_), and by increasing K^+^ currents β-adrenoceptor antagonists cause negative chronotropy. Their classification is based on their chemical structure, βselectivity and pharmacokinetic properties. There are two subtypes of both α- and β-adrenoceptors. They have distinct distribution and density in different tissues. β_1_-receptors have a more intense presence in the heart and β_2_-receptors have a higher density in the smooth muscles and in the bronchi, however α1-, β_1_-, and β_2_-adrenoceptors occur on the surface of the cardiomyocytes. β-adrenoceptor antagonists mainly act on β_1_-and β_2_-adrenoceptors (Brodde, 2007; Schnee et al., 2008). Moreover, some of them have an intrinsic sympathomimetic activity caused by a partial β-agonist property, and others have vasodilator effects due to an associated α-blocker feature (Opie and Yusuf, 2001; Dorian, 2005). First generation β-adrenoceptor antagonists (such as propranolol, timolol, nadolol) inhibit β_1_- and β_2_-adrenoceptors equally, thus their antiarrhythmic effect is limited. Second generation of β-adrenoceptor antagonists (e.g., metoprolol, bisoprolol, atenolol, etc.) has a more pronounced β_1_-selectivity especially in low doses. In this group bisoprolol is known as the most cardioselective agent with explicit antiarrhythmic activity. Third generation β-adrenoceptor antagonists (e.g., labetalol, carvedilol, nebivolol) has an additional vasodilating effect, hence they are usually ordered as antiarrhythmics mainly in hypertensive patients (do Vale et al., 2019). As effective antiarrhythmics, β-adrenoceptor antagonists decrease the mortality of patients with myocardial infarction by more than 30% (Norris et al., 1984; Teo et al., 1993; Hjalmarson, 1999). In the case of CPR and refractory VF, β-adrenoceptor antagonists (e.g., esmolol) were also reported to have moderating effect on the proarrhythmic property of epinephrine. Although success has been reported in relation to the administration of esmolol, considering the limited number of cases there are no straightforward recommendations regarding its use yet (Lee et al., 2016). In a study published in 2007, Bourque et al. summarized the results on the application of β-adrenoceptor antagonists. They presented the findings of experiments conducted between 1966 and 2006 on dogs and rats primarily, where the more favorable effects – compared to epinephrine – of β-adrenoceptor antagonists on myocardial oxygen demand were found among others (Bourque et al., 2007).

β_1_-and β_2_-adrenoceptors are also blocked by sotalol, however it also inhibits the rapid component of delayed rectifier potassium currents leading to a prolonged ventricular repolarization. Due to its combined mode of action, the racemic sotalol can be classified as a type II + III antiarrhythmic drug. The two enantiomers of sotalol display different pharmacodynamic activities and selectivities: D-sotalol blocks dominantly the rapid component of the delayed rectifier potassium current (I_Kr_), while L-sotalol inhibits both I_Kr_ current and β-adrenoceptors (Funck-Brentano, 1993). In the SWORD trial, which had to be terminated prematurely due to the increased occurrence of ventricular arrhythmias, administration of D-sotalol was associated with a higher mortality rate among 3,121 patients with left ventricular EF ≤40% after myocardial infarction (Waldo et al., 1996). Therefore, D-sotalol is not used as an antiarrhythmic drug in the everyday clinical practice. D,L-sotalol (racemic sotalol containing equimolar amount of D and L enantiomers) inhibits mainly β-adrenoceptors and is appropriate for the treatment of malignant ventricular arrhythmias, especially in conditions with elevated serum catecholamine levels or increased sensitivity for catecholamines e.g. in the case of acute myocardial infarction, phaeochromocytoma, mitral prolapse, postoperative arrhythmias, and thyreotoxicosis. However, sotalol’s torsadogenic effect limits its applicability (Opie and Yusuf, 2001). Although several animal experiments have been carried out to investigate the effect of sotalol on ventricular fibrillation so far, further data are needed to form recommendations in this indication (Jin et al., 2018). In the 2015 ESC Guideline for ventricular arrhythmias and SCD the application of sotalol is recommended for patients with coronary artery disease, but only in the case of implanted ICD due to its proarrhythmic effect (Priori et al., 2015).

According to our knowledge quinidine can play a life-saving role in patients with BrS, ERS, and idiopathic ventricular fibrillation (IVF). Quinidine is a class IA antiarrhythmic drug, that blocks α-adrenoceptors, muscarinic acetylcholine receptors and several types of voltage gated K channels beyond Kv1.5 Na channel. It reduces the frequency of impulse generation by prolonging spontaneous diastolic depolarization and elevates the threshold potential of action potentials. Oral quinidine is recommended for patients with ICD due to BrS, ERS, or IVF, while it decreases the number of ICD shocks and it also improves ICD-free survival (Malhi et al., 2019).

Amiodarone is an iodinated benzofuran, which was originally developed for the treatment of angina pectoris. Amiodarone prolongs the action potential duration and increases the refractory period of the atrial and ventricular myocardium, the AV-node and the Purkinje system (Mason, 1987). Amiodarone, a type III antiarrhythmic drug, having the qualities of all the four groups of antiarrhythmic agents-, blocks the activity of sodium and potassium channels, antagonises the functioning of both α- and β-adrenergic receptors and as a mild calcium antagonist it also has a blocking effect on the sinoatrial node, and the AV-nodal tissue (Lubic et al., 1994; Sampson and Kass, 2011). In the case of VF recurring after three defibrillations, the administration of 300 mg and later 150 mg of amiodarone is recommended (Neumar et al., 2015) It was based on the findings of two major studies, conducted in 1999 and 2002, that amiodarone replaced the earlier recommended lidocaine. In 2016, Laina et al. analyzed the findings of 1,663 studies conducted with amiodarone and found that amiodarone significantly increased short-term survival rate (OR = 1.42 p = 0.015). Findings with reference to the long-term effects of amiodarone affecting the neurological outcome primarily are not straightforward, although encouraging when compared to other antiarrhythmic agents (Laina et al., 2016; Karlis and Afantenou, 2018). In 2018, American Heart Association (AHA) recommended the further application of amiodarone or lidocaine in the case of VF/pVT due to their short-term positive effects (Panchal et al., 2018). The ROC-ALPS study investigated the short and long-term effects of captisol-based formulation of amiodarone and lidocain in the case of out-of hospital cardiac arrests. Both had a better short-term effect compared to the placebo, however, there have been no differences shown regarding long-term outcome, neurological status, and admission from hospital (Kudenchuk et al., 2016).

A synthetic amiodarone analogue, dronedarone is not iodinated, as it has been designed for avoiding the side effects of amiodarone. Dronedarone blocks L-type Ca, K, and Na channels. In animal studies the antiarrhythmic effect of dronaderone on the ventricular myocardium has been described (Finance et al., 1995). Additionally, in three human studies, it significantly decreased ventricular arrhythmogeneity, and the number of ICD shocks (Fink et al., 2011). However, in lack of widespread experiences it is still not applicable for the prevention and treatment of VF.

Taking all the available data into consideration it can be concluded that currently there is no appropriate antiarrhythmic agent that can safely prevent VF and would significantly improve the long-term outcome of patients with life-threatening ventricular arrhythmias (Panchal et al., 2018).

### Considerations for the Treatment of Refractory Ventricular Fibrillation

In the case of refractory VF (refibrillation), the resuscitation recommendation suggests administering shocks of growing intensity up to 360 J (Soar et al., 2015). In case the ALS protocol fails, some authors recommend dual defibrillation (dual-sequential defibrillation [DSD]). In the latter case a second pair of electrodes in addition to the traditional pair is placed in an anteroposterior position and accordingly shock is administered with two defibrillators. The vector of the electric impulse administered changes compared to the original, which enables the defibrillation of larger parts of the cardiac muscle and may be more effective in the case of corpulent patients as well. In a retrospective study of 4 years, Cortez et al. found, out of 2,428 out-of-hospital circulatory collapses 12 cases of refractory VF where the application of the ALS protocol failed, the applied DSD was successful in 3 cases (ROSC and good CPC value) (Cortez et al., 2016). In order to enhance the chance of success Frye et al. combined the DSD protocol with administering lidocaine, as did the authors of other case studies as well (Frye et al, 2018). Although the ERC recommendation currently in force advises the administration of lidocaine during resuscitation only in the case of recurring VF or pregnancy, there are increasing attempts for using it in the case of refractory VF as well (Priori et al., 2015). While there is a wider use of devices, it is a disadvantage of DSD that uniform protocols are not available and there are no comprehensive large-scale studies on patients who have undergone DSD (Frye et al., 2018).

## Long-Term Treatment After Aborted Cardiac Death

**Figure 3:**
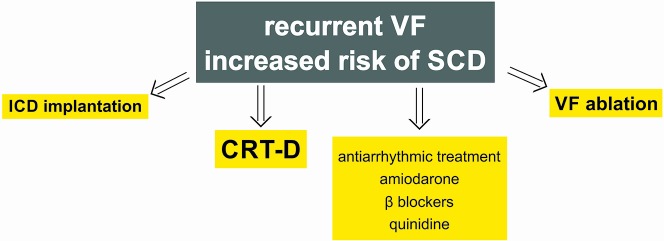
Long-term therapy of ventricular fibrillation. ICD, implantable cardioverter defibrillator; SCD, sudden cardiac death; VF, ventricular fibrillation; CRT-D, cardiac resynchronization therapy combined with ICD.

Regarding long-term treatment of malignant ventricular arrhythmias, the application of implantable cardioverter defibrillator (ICD) is an important element, which releases an antitachycardia pacing or an electric shock at the occurrence of the rhythm disturbance (**Figure 3**).

In a study with 2,521 patients involved Bardy et al. found that the implantation of an ICD able to deliver only shocks, combined with conventional conservative therapy, reduced total mortality rate by 23% in the case of NYHA II and III heart failure patients with EF <35% compared to conservatively treated patients (Bardy et al., 2005). Antitachycardia pacing (ATP) is in fact meant to reduce the number of superfluous or inappropriate shocks, whereby patients’ quality of life improves, and the lifetime of the device also grows. Most often the device delivers superfluous shocks due to supraventricular arrhythmias, which occurred in 8%–40% of the patients despite appropriate drug therapy. Moreover, ATP is also successful in stopping slow and fast ventricular tachycardia in almost 85%–90% of the cases, although it may happen in both cases if timing or setting are inappropriate that the ATP itself generates a malignant ventricular arrhythmia (Fisher et al., 1978; Schuger et al., 2012). In the CASCADE study cardiac dysrhythmias due to ICD dysfunction have been shown to be significantly reduced by long-term administration of amiodarone (CASCADE study, 1991). This favourable effect of amiodarone is more pronounced compared to class IC antiarrhythmic drugs, e.g., quinidine, procainamide, and flecainide (Huang et al., 1991). However, there are cases reporting the effectiveness of long-term quinidine therapy administered shortly after intravenous infusion of β-adrenoceptor agonist isoproterenol in the case of VF storms in patients with BrS, when amiodarone was ineffective (Jongman et al., 2007; Dakkak et al., 2015). According to further observations the number of appropriate and inappropriate ICD shocks can be reduced by sotalol, nevertheless overall mortality seems not to improve compared to placebo (Kühlkamp et al., 1999; Bollmann et al., 2005). Azimilide, another class III antiarrhythmic drug equally inhibits the rapid and slow components of delayed rectifier K^+^ currents. It has the same positive effects as amiodarone, but it is known to cause less proarrhythmic effect. Additionally, azimilide has not been proven to reduce left ventricular systolic function during long-term antiarrhythmic treatment (Dorian et al., 2004). Positive effects of dronaderone and dofetilide (blocker of the rapid component of delayed rectifier K^+^ current) were also described, but less antiarrhythmic and mortality reducing effects were verified compared to amiodarone and sotalol (Bollmann et al., 2005). Recurrent VF episodes were registered in more than one third of ICD patients treated with amiodarone alone. Importantly, more favorable results were found in patients on combined β-adrenoceptor antagonist and amiodarone management (Connolly et al., 2006).

Newest ICDs are suitable for the purpose of cardiac resynchronization therapy (CRT) as well. Tang et al. examined 1798 patients, half of whom were grouped, in a randomized way, into the category ‘only ICD’, while the other half into the category ICD complemented by CRT. In the course of the 40-month examination period they found that the relative risk of cardiovascular death in the CRT group fell by 24% compared to the ‘only ICD’ group, and the frequency of hospitalization was also signifcantly lower (Tang et al., 2010).

The European Society of Cardiology has issued recommendations for subcutaneous ICDs as well. The malfunctioning of these devices develops rarely, although the application of ATP or cardiac resynchronization is not available.

The implantation of ICD’s is mainly performed as secondary prevention combined with β-adrenoceptor antagonist therapy (e.g. metoprolol 100 mg/die, carvedilol 50 mg/die or bisoprolol 10 mg/die) for patients who suffered malignant ventricular arrhythmia episodes (Connolly et al., 2006). For the purpose of primary prevention ICD’s are implanted primarily for patients with congenital heart diseases, but according to the AHA protocol currently in force it is to be considered for patients suffering from heart failure as well (Al-Khatib et al., 2018). MADIT, MADIT-II, and SCD-HeFT trials proved that populations suffering from heart failure and ischemic heart disease, ICD implanation significantly improved overall mortality without any additional therapies (Moss et al., 1996; Moss et al., 2002; Mark et al., 2006). Accordingly, ICD implantation as a primary prevention is recommended for NYHA I heart failure patients where left ventricular ejection fraction is under 30%, for NYHA II and III patients where EF is under 35% and in the case of ischemic heart disease where EF is less than 40% (Al-Khatib et al., 2018).

The administration of β-adrenoceptor antagonists has especially great significance in the case of congenital heart disease patients and primary ion channel diseases. Both the short- and long-term survival of inherited long QT syndrome patients is clearly positively influenced β-adrenoceptor antagonist therapy unless it is the LQT3 type. The suggested β-adrenoceptor antagonists are propranolol (2–4 mg/kg/die) or the long-acting nadolol (1.5 mg/kg/die), while other β-adrenoceptor antagonists, e.g., metoprolol or atenolol seem to be ineffective in this particular indication. Regarding LQT3, drugs decreasing the late sodium current, e.g., flecainide, dofetilide, mexiletine, or ranolazine can be administered (Schwartz et al., 2012; Antzelevitch et al., 2014). Wilde et al. examined 118 LQT3-type LQT syndrome patients who had suffered some cardiac event (SCD, syncope, aborted cardiac death). They found that the administration of β-adrenoceptor antagonist reduced the development of a similar cardiac event among these patients later on by 83% in the case of women, while no such difference was found in the case of men (Wilde et al., 2016). The study of Shimizu et al. also emphasises the difference between the two sexes. They sequenced the clinical genomes of 1,124 patients including the pathological mutations causing the long QT syndrome. The retrospective data and the genetic findings together highlight the fact that LQT1 and LQT2 mutations much more frequently involve malignant ventricular arrhythmias in the case of women, while as regards the other 3 major types of mutation no differences can be detected between the two sexes (Shimizu et al., 2019). This is one reason why a widening range of attempts have been made for the genetically based risk assessment of channelopathies as it may be of use in the selection of closer observation and specific drug therapy.

ACE-inhibitors and aldosterone antagonists (eplerenone), which protect from ventricular arrhythmias associated to ischemic heart disease primarily, reduce the heterogeneity of APD and left ventricular reverse remodelling and lower the risk of sudden cardiac death (Alberte and Zipes, 2003; Pitt et al., 2003). It was confirmed in the CONSENSUS study already that in the period examined the administration of enalapril in the case of 253 patients suffering from NYHA IV heart failure reduced the development of SCD by 27% (CONSENSUS Trial Study Group, 1987). In the SOLVD study examining 2569 patients Lam et al. found that the administration of 20 mg enalapril per day reduced overall mortality by 12% and cardiovascular mortality by 4% in the case of heart failure patients (Lam et al., 2018). In the SAVE trial on 2,231 patients the administration of 3x25 mg captopril daily reduced overall cardiovascular mortality by a few percent compared to the placebo group but it did not meaningfully affect the risk of sudden cardiac death (Pfeffer et al., 1992). In the HOPE study 9,297 patients with preserved left ventricular function, but high cardiovascular risk were examined. Patients taking 10 mg of ramipril daily had a relative risk of 0.62 for SCD compared to placebo group (The Heart Outcomes Prevention Evaluation Study (HOPE) Investigators, 2000). Based on PROGRESS, EUROPA and ASCOT-BLPA trials the administration of 4–10 mg perindopril daily (individual dose is required based on kidney function and other taken drugs) significantly reduced cardiovascular mortality and the occurence of sudden cardiac death (Campbell, 2006). On the contrary, in the IMAGINE study 2,553 patients with left ventricular ejection fraction ≥40% were examined who underwent CABG, where 40 mg quinapril were given daily early after the operation. Compared to the placebo group there were no significant positive effects on cardiovascular mortality and on the occurrence of ventricular arrhythmias up to 3 years after surgery. However, ACE-I treatment increased the incidence of adverse events, mainly early after CABG (Rouleau et al, 2008).

The angiotensin-receptor blocker candesartan was proven, based on the CHARM low LVEF study published in 2004, to signifcantly reduce overall mortality as well as cardiovascular mortality when added to the base therapy of chronic heart failure patients with left ventricular EF of lower than 40% (Young et al., 2004). The same was confirmed by the favorable findings of the RESOLVD study where candesartan was added to the ACE-inhibitor (enalapril) and β-adrenoceptor antagonist (metoprolol) and the triple combination produced more favorable prognosis (McKelvie et al., 2003). Based on the data of ONTARGET, PROFESS and TRANSCEND studies the positive effects of angiotensin receptor blocker telmisartan at the daily dose of 80 mg on cardiovascular mortality and on the occurence of ventricular arrhythmias were confirmed. At the same time the superior effects of ACE-Is (e.g., ramipril, perindopril) compared to telmisartan were also noted (DiNicolantonio and O’Keefe, 2014).

Statins are inhibitors of the hydroximethylglutaryl coenzyme A reductase, i.e., they reduce the serum cholesterole level, in addition to which, thanks also to their pleiotropic effect, they reduce the likelihood of malignant ventricular arrhythmias. The DEFINITE study examined 229 non-ischemic heart disease patients undergoing ICD therapy where the administration of statins significantly reduced ventricular arrhythmogenesis and sudden cardiac death compared to patients without statin administration (Goldberger et al., 2006).

Furthermore, in the case of arrhythmias triggered by EADs, inhibitors of the L-type Ca^++^ currents may be administered as part of the long-term therapy. Morita et al. found that the late type Na channel blocker ranolazine reduced repolarization heterogeneity, and increased the threshold potential value required for the development of VF. In addition, its non-selective blocking property on L-type Ca channels and I_Kr_, I_Ks_ currents was detected, too. Ranolazine is also effective in the case of VF with underlying reentry mechanism (Morita et al., 2011) and has been furthermore proven to signifcantly reduce the delivery of inappropriate ICD shocks (Bunch et al., 2011; Zareba et al., 2018). However, further data are needed to clearly evaluate the exact role of ranolazine in the prevention of SCD. According to the latest ESC guideline, ranolazine is recommended only in the case of LQTS3 patients as a preventive treatment of ventricular fibrillation (Priori et al., 2015).

The cyclin-dependent kinase inhibitor roscovitine, originally developed as a chemotherapeutic agent, accelerated the inactivation of L-type Ca channels in the course of model experiments, thereby reducing the likelihood of the development of ventricular arrhythmias (Krummen et al., 2016). Further research is however needed with reference to these agents.

## Ablation of VF Triggers and Substrates

In the cases of recurrent or refractory VF and ICD storms secondary to repeated VF episodes, ablation of VF triggers and substrates have to be taken into consideration. In these circumstances electrophysiological testing, and intracardiac mapping are performed (Cheniti et al., 2017). In order to clearly localize the ablation site, beyond classical pace mapping, entrainment and phase mapping, lately, activation, or substrate mapping have been accomplished (deBakker, 2019). Radiofrequency ablations of both VF triggers and subtrates have to be achieved. Endocardial data can be completed with epicardial maps served by electrocardiographic imaging (ECGI) method. During ECGI process a special vest with 252 surface electrodes is worn by the patients. Data of ECGI combined with computer tomography raise the opportunity of a more exact localization of arrhythmia foci. During ECGI procedure together with intracardiac mapping, both endo- and epicardial maps can be gained by the investigator (Singh and Noheria, 2018).

In VF patients with ischemic heart disease, non-ischemic cardiomyopathies, valvulopathies, amyloidosis, long QT syndromes and IVF, short-coupling triggering ventricular premature beats originating from the Purkinje system were identified and ablated (Cheniti et al., 2018; Singh and Noheria, 2018). In 2011, successful epicardial ablations of premature beats of the right ventricular outflow tract (RVOT) were published in patients with BrS. In 2019, another study provided data on the VF ablation of 52 ERS patients. These interventions were carried out in the right ventricle, RVOT and Purkinje fibres. In both studies, during a 3-year follow-up period, 90% of patients were reported to be free of recurrent VF episodes (Nademanee et al., 2011; Nademanee et al., 2019).

## Summary

Sudden cardiac death is a leading death cause in developed countries, its fast and effective treatment as well as prevention are therefore high priority tasks. There are several non-invasive diagnostic opportunities serving the timely detection of diseases increasing susceptibility to inherited and acquired ventricular arrhythmia, and earliest possible detection is followed, as part of a long-term therapeutic strategy, by the assessment and application of drug and device therapy on an individual basis. In the case of cardiopulmonary resuscitation early defibrillation in addition to minimally interrupted chest compression has been proven to improve the outcome of patients. Radiofrequency ablation of VF triggers and substrates is also a feasible and emerging therapeutical opportunity.

## Author Contributions

All authors confirm that they have read and approved the paper and they have met the criteria for authorship. ZS: wrote the manuscript. DU: took part in preparing the manuscript. TÖ: took part in preparing the manuscript. VS: took part in preparing the manuscript. PN: prepared and reviewed the manuscript before publication.

## Funding

This work was funded by GINOP-2.3.2-15-2016-00062.

## Conflict of Interest

The authors declare that the research was conducted in the absence of any commercial or financial relationships that could be construed as a potential conflict of interest.
